# Supplementary material for: Saccharomyces boulardii (CNCM I-745) improves intestinal damage in sepsis by remodeling intestinal flora structure
Source: Front Cell Infect Microbiol. 2025 Jul 31;15:1602792. doi: 10.3389/fcimb.2025.1602792 (PMC12350329; doi:10.3389/fcimb.2025.1602792)
Supplement: Supplementary file 1 [file DataSheet1.zip › summary/summary/7_Differential_analysis/2_LEfSe_result/Group_AW4_vs_AW0_0.05_3.0/LEfSe_Group_AW4_vs_AW0.barplot.pdf]

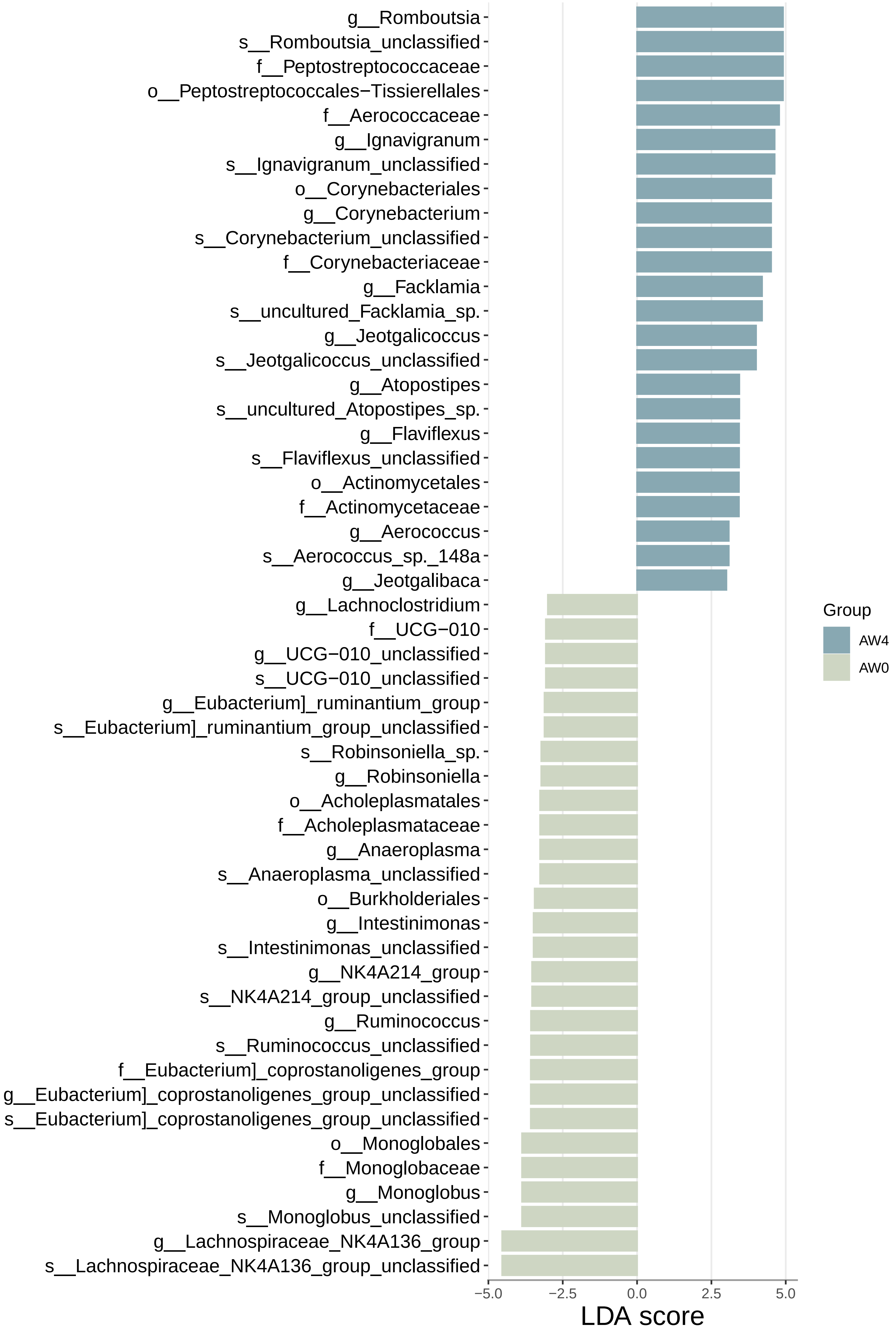
g__Romboutsia
s__Romboutsia_unclassified
f__Peptostreptococcaceae
o__Peptostreptococcales−Tissierellales
f__Aerococcaceae
g__Ignavigranum
s__Ignavigranum_unclassified
o__Corynebacteriales
g__Corynebacterium
s__Corynebacterium_unclassified
f__Corynebacteriaceae
g__Facklamia
s__uncultured_Facklamia_sp.
g__Jeotgalicoccus
s__Jeotgalicoccus_unclassified
g__Atopostipes
s__uncultured_Atopostipes_sp.
g__Flaviflexus
s__Flaviflexus_unclassified
o__Actinomycetales
f__Actinomycetaceae
g__Aerococcus
s__Aerococcus_sp._148a
g__Jeotgalibaca
g__Lachnoclostridium
f__UCG−010
g__UCG−010_unclassified
s__UCG−010_unclassified
g__Eubacterium]_ruminantium_group
s__Eubacterium]_ruminantium_group_unclassified
s__Robinsoniella_sp.
g__Robinsoniella
o__Acholeplasmatales
f__Acholeplasmataceae
g__Anaeroplasma
s__Anaeroplasma_unclassified
o__Burkholderiales
g__Intestinimonas
s__Intestinimonas_unclassified
g__NK4A214_group
s__NK4A214_group_unclassified
g__Ruminococcus
s__Ruminococcus_unclassified
f__Eubacterium]_coprostanoligenes_group
g__Eubacterium]_coprostanoligenes_group_unclassified
s__Eubacterium]_coprostanoligenes_group_unclassified
o__Monoglobales
f__Monoglobaceae
g__Monoglobus
s__Monoglobus_unclassified
g__Lachnospiraceae_NK4A136_group
s__Lachnospiraceae_NK4A136_group_unclassified
−5.0
−2.5
0.0
2.5
5.0
LDA score
Group
AW4
AW0
